# Associations among frailty conditions and pain indicators: Data from 22 356 European older adults

**DOI:** 10.1111/ggi.15016

**Published:** 2024-11-06

**Authors:** Trinidad Sentandreu‐Mañó, Elena Marques‐Sule, Pallav Deka, José M Tomás, Leiver Andrés Romero Pintado, Leonie Klompstra, Hady Atef

**Affiliations:** ^1^ Department of Physiotherapy University of Valencia Valencia Spain; ^2^ Physiotherapy in Motion, Multispeciality Research Group (PTin MOTION), Department of Physiotherapy University of Valencia Valencia Spain; ^3^ College of Nursing Michigan State University East Lansing MI USA; ^4^ Department of Methodology for the Behavioral Sciences University of Valencia Valencia Spain; ^5^ Department of Health, Medicine and Caring Sciences Linkoping University Linkoping Sweden; ^6^ School of Allied Health Professions (SAHP) Keele University Staffordshire UK; ^7^ Department of Physical Therapy for Cardiovascular/Respiratory Disorders and Geriatrics, Faculty of Physical Therapy Cairo University Cairo Egypt

**Keywords:** aging, Europe, frailty, older adults, pain

## Abstract

**Aim:**

Recent studies supported the presence of a relationship between pain and frailty, but more research is needed to highlight the pain–frailty association. The study aimed to investigate the prevalence and the influence of different pain indicators on frailty while controlling for age, sex and country.

**Methods:**

This observational study used data from the sixth wave of the SHARE survey. A sample of 22 356 community‐dwelling individuals aged >60 years from six European countries (Spain, Estonia, France, Greece, Czech Republic and Sweden) was analyzed. The pain was measured through assessment of medication used for joint pain or other types of pain, pain location, polypharmacy and pain level. Frailty was assessed with the modified Fried Frailty phenotype.

**Results:**

Pain indicators, especially widespread pain and pain severity, were significantly associated with prefrailty (odds ratio 3.30, 95% CI 2.40, 4.55; and odds ratio 0.61, 95% CI 0.51, 0.72) and frailty status (odds ratio 4.69, 95% CI 3.31, 6.67; and odds ratio 0.37, 95% CI 0.30, 0.44). Advancing age and female sex consistently correlated with increased prefrailty (odds ratio 1.06, 95% CI 1.05, 1.07; and odds ratio 1.36, 95% CI 1.22, 1.53) and frailty risk (odds ratio 1.11, 95% CI 1.10, 1.12; and odds ratio 1.71, 95% CI 1.48, 1.96). Country‐specific differences emerged, with Spaniards showing higher odds ratios of prefrailty and frailty compared with Swedish, French and Czech individuals, whereas Greeks showed elevated odds ratios compared with Spaniards. The factors associated jointly explained 27.5% of the variance in frailty categories.

**Conclusion:**

Significant associations were identified, particularly with widespread pain and pain severity, highlighting their impact on frailty. Country‐specific variations in frailty prevalence were observed, alongside consistent associations with advancing age and female sex. These findings provide valuable insights into the intricate interplay between pain and frailty, offering the potential for targeted interventions in older adults' care through tailored pain management strategies. **Geriatr Gerontol Int 2024; 24: 1362–1369**.

## Graphical abstract

Significant associations between frailty and pain indicators were found in older adults across Europe, varying depending on the specific indicator. Pain severity and widespread pain are notably related to prefrailty and frailty status. Differences in frailty prevalence by country, age and sex highlight their influence on the pain–frailty relationship.

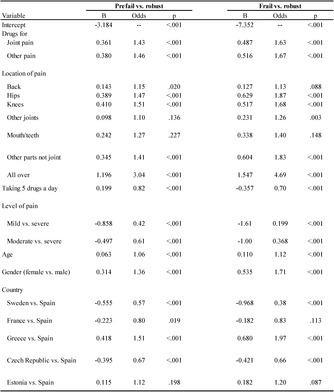

## Introduction

The increase in life expectancy over the past few decades has produced significant growth in the older adult population. It is estimated that by 2050, almost one‐third of the world's population will constitute people aged >65 years. The number of persons aged ≥80 years is expected to triple between 2020 and 2050 to reach 426 million.^1^, ^2^

Aging is a complex and inevitable process that is associated with pathophysiological changes, such as altered perception of pain, loss of muscle mass, loss of strength and frailty.^3^, ^4^ These changes lower functional capacity in older adults and create greater dependence on caregivers,^5^ increasing the cost of care and burden on public health.^6^

Pain is defined as an unpleasant sensory and emotional experience associated with actual or potential tissue damage or disease.^7^, ^8^ Pain experience is a common problem in aging, and its management has significant clinical impact.^9^ In other words, chronic joint pain is related to poorer health status, poor physical function, greater use of medications (polypharmacy) and greater disability.^10^, ^11^, ^12^, ^13^ Alterations in the pain processing systems with age can impact pain perception and threshold, thereby inducing a differential experience of pain in older adults.^13^ The alterations in pain perceptions increase the frequency of accidents and, indirectly, contribute to a higher prevalence of pain. Additionally, the assessment of pain is complicated because of the presence of multiple comorbid conditions or polypharmacy.^14^, ^15^

Frailty is defined as an increase in vulnerability to stressors produced by an alteration in several interrelated systems that leads to a decrease in the homeostatic reserve and adaptability, which predisposes an individual to adverse health events.^16^, ^17^ Frailty is associated primarily with older adults, adversely impacts health outcomes, and often leads to disability, institutionalization and death.^17^ Frailty can manifest as a result of malnutrition, advancing age, weight loss, weakness or polypharmacy.^18^ Frail individuals have a greater probability of falls, often as a result of impaired balance and gait.^19^ Frailty is a strong predictor of geriatric syndromes, such as falls, loss of mobility and cardiovascular disease, among the aging population.^20^, ^21^

Recent studies have shown that the multifactorial nature of pain and frailty in older adults makes its management challenging.^5^, ^9^, ^17^ Chronic widespread pain in older adults is associated with the development or worsening of frailty.^22^ Additionally, a longitudinal study showed that the presence of chronic pain, but not intrusive pain, increased the risk of developing physical frailty in older Australian men.^23^ Given its multidimensional nature,^24^ pain affects the various physiological systems, decreasing energy reserves and impairing homeostasis,^9^, ^17^ a phenomenon referred to as “pain homeostasis.”^24^ Older adults with multiple comorbidities and experiencing pain are likely to be less active and functional, and are at higher risk of developing frailty. In particular, a recent study found that pain was linked to frailty independently of painful and non‐painful morbidities, underscoring the need for further research to understand the complex relationship between both conditions.^25^

Pain and frailty have a great impact on older adults. The current literature indicates that there is an association between pain and frailty status, but the physiological mechanisms, links or causes are not fully understood.^22^, ^26^ In contrast, it seems that all pain indicators are not equally associated with frailty, given the controversial results in the literature. There is limited evidence analyzing the relationships between various characteristics of pain within the context of a frailty model. Therefore, there is a need to gather evidence on different indicators of pain management and their associations with the physical dimension of frailty. All in all, we hypothesize that specific characteristics of pain might show differential associations with frailty levels, independent of sociodemographic and geographic factors. Therefore, the present study aimed to: (i) explore the presence of pain indicators and frailty in a sample of older adults from different European countries; and (ii) deepen the understanding of which pain indicators (pain medication, pain location, polypharmacy and pain level) have the greatest association with frailty levels, while considering for potential confounding variables, such as age, sex and country. To our knowledge, this is the first study to investigate these links between pain and frailty among European older adults to provide insights that could contribute to the advancement of more informed and comprehensive approaches toward developing patient‐centered and individually tailored treatments.

## Methods

### Design and participants

A cross‐sectional study was carried out using data from the sixth wave of the Survey of Health, Ageing and Retirement in Europe (SHARE). This project is a longitudinal study of the European Union based on probabilistic sampling that aims to study people aged >50 years, who reside in different European countries.^27^ The fieldwork of this wave was completed in November 2015. In all waves, most of the data were collected by interviewers using computer‐assisted personal interviewing.^27^ The SHARE target population included individuals who were aged ≥50 years old at the time of sampling and who resided regularly in the respective SHARE country. Exclusions were made for those who were incarcerated, hospitalized or abroad for the entire duration of the survey, as well as for those who could not speak the countries' languages, could not be located due to errors in the sampling frame (such as non‐existent addresses or vacant homes) or had moved to an unknown address.^28^ For this study, a sample of 22 356 individuals aged ≥60 years belonging to six European countries was analyzed. These countries were selected based on geographical location, trying to cover countries from each region of Europe: Sweden from the north; the Czech Republic from the center; Greece from the south; Spain and France from the southwest, and Estonia from the northwest. The SHARE project has been approved by the Ethics Committee of the Max Planck Society for the Advancement of Science, and collects information related to health, socioeconomic status and socio‐family context.

### Outcomes and instruments

The following pain indicators have been used in this study: pain medication, pain location, polypharmacy and pain level.

The pain medication variable is the sum of two variables, medication for joint pain and medication for other types of pain: (i) medication for joint pain was recorded using the question “Do you currently take medication at least once a week for joint pain?” The response was coded as “yes” or “no”; and (ii) to record medication for other types of pain, the following question was used: “Do you currently take medication at least once a week for other pain?” The response was coded as “yes” or “no.”

The pain location included various areas, each with its respective question. The response of each was coded as “yes” or “no”: back: “Do you have pain in your back?”; hip: “Do you have pain in the hip?”; knees: “Do you have pain in your knees?”; other joints: “Do you have pain in other joints?”; mouth/teeth: “Do you have pain in your mouth or teeth?”; other parts of the body that are not joints: “Do you have pain in other parts of the body that are not joints?”; and widespread: “Do you have pain everywhere?”

Polypharmacy was recorded using the following question “Do you take at least five different medications on a typical day? Include medications prescribed by your doctor, medications you buy without a prescription and dietary supplements, such as vitamins and minerals.” The response was coded as “yes” or “no.”

Pain level was recorded using the following question: “How bad is the pain most of the time?” The possible answers were: mild, moderate or severe.

Frailty was defined using the Fried phenotype, which is made up of five physical criteria: involuntary weight loss of >10 pounds (4.54 kg) for >1 year; weakness; tiredness; slowness or slow walking; and low level of physical activity. In the present study, the same indicators used by Santos‐Eggimann *et al*. for which adaptations of the criteria were made to the contents of the SHARE survey that was carried out previously.^29^ The criteria were recorded as follows: involuntary weight loss, weakness, fatigue, slowness and low level of physical activity.

Involuntary weight loss was recorded in two ways. By reporting a “decreased desire for food” in response to the question “How has your appetite been?” or, by answering “less” to the question “Have you been eating more or less than usual?” Weakness was recorded from the highest value of the four consecutive measurements of grip strength that were made using a dynamometer, making two in each hand and applying the limits of sex and body mass index established by Fried. Fatigue was measured using the following question: “In the last month, have you had little energy to do the things you wanted to do?” with a dichotomous answer of “yes” or “no.” Tiredness was recorded when a “yes” answer was given to this question. Slowness was recorded by a positive response to the following question: “Due to health problems, do you have difficulty walking 100 m or climbing a flight of stairs without resting?” The difficulties were expected to last >3 months. For the low level of physical activity, the following question was asked: “How often do you engage in activities that require a low or moderate level of energy, such as gardening, cleaning the car or going for a walk?” This criterion was fulfilled when the participants answered “one to three times a month,” “hardly ever” or “never.”

When classifying, each fulfilled criterion was equivalent to one point, following the guidelines of Fried *et al*.^21^ Those with zero points were classified as “robust or not frail,” those with 1 or 2 points as “prefrail,” and those with 3–5 points were classified as “frail.”

### Statistical analysis

Descriptive statistics of all variables were carried out, including the case of quantitative variables statistics of central tendency and variability, and frequencies (absolute and relative) in the case of categorical variables. Additionally, bivariate associations among the variables were calculated with Spearman's rho coefficients. Finally, multinomial logistic regression has been estimated to associate in a multivariate context frailty (categories) with all indicators of pain while controlling for age, sex and country of residence. These potential confounding variables have been previously related to pain, frailty or both (see for example^25^, ^30^, ^31^). The standard SPSS procedure to handle with missing data in SPSS, listwise deletion, was used (IBM, Armonk, NY, USA).

## Results

### Sample descriptive statistics

The sample included 22 356 individuals. The final sample size comes from the original 68 231 participants in wave 6, after applying the inclusion criteria: reducing the sample to include only those participants aged ≥60 years, and being sampled in the selected countries. Of these, 7744 (34.6%) were classified as robust, 10 985 (49.1%) as prefrail and 3627 (16.2%) as frail. The mean age was 71.96 ± 8.27 years, with a maximum age of 105 years and a minimum of 60 years.

The sample included more women (56.3%) than men. The majority of the participants were from Spain (20.1%), and were married and living with their spouse (64.7%). The average years of education were 10.48 ± 4.32, with a maximum of 25 years of education and a minimum of 0 years. In addition, on average participants had two comorbid conditions with the highest being 12. The sociodemographic characteristics of the sample are represented in Table 1. The frequencies (absolute and relative) of the pain indicators according to the frailty classification are also shown in Table 2.

**Table 1:** Sociodemographic characteristics of the sample

Variable	Mean ± SD or *n* (%)
*N*	22 356
Robust	7744 (34.6)
Prefrail	10 985 (49.1)
Frail	3627 (16.2)
Country	
Sweden	3397 (15.2)
Spain	4497 (20.1)
France	2861 (12.8)
Greece	3558 (15.9)
Czech Republic	3896 (17.4)
Estonia	4147 (18.5)
Age (years)	71.96 ± 8.27
Sex	
Male	9766 (43.7)
Female	12 590 (56.3)
Marital status	
Married, living together with spouse	14 316 (64.7)
Registered relationship	231 (1.0)
Married, living apart from spouse	240 (1.1)
Never married	1036 (4.7)
Divorced	1735 (7.8)
Widow	4581 (20.7)
Years of education	10.48 ± 4.32
No. comorbid conditions	2.06 ± 1.65

SD, standard deviation.

**Table 2:** Absolute and relative frequencies of pain indicators according to the frailty classification (robust, prefrail and frail)

Variables of pain	Robust, *n* (%)	Prefrail, *n* (%)	Frail, *n* (%)
Joint pain medications	622 (8.1%)	2630 (24%)	1526 (42.2%)
Medications for other types of pain	638 (8.3%)	2376 (21.7%)	1311 (36.3%)
Pain location			
Localized back pain	1186 (48%)	2926 (47.6%)	1318 (48%)
Localized pain in the hips	379 (15.3%)	1498 (24.3%)	865 (31.5%)
Localized pain in the knees	795 (32.2%)	2600 (42.3%)	1299 (47.4%)
Localized pain in other joints	724 (29.3%)	1971 (32%)	1018 (37.1%)
Localized pain in the mouth/teeth	46 (1.9%)	144 (2.3%)	74 (2.7%)
Localized pain in parts of the body other than joints	434 (17.6%)	1220 (19.8%)	647 (23.6%)
Widespread pain	61 (2.5%)	498 (8.1%)	323 (11.8%)
Polypharmacy	806 (14.8%)	3123 (32.1%)	1954 (55.7%)
Pain level			
Mild	930 (37.6%)	1211 (19.7%)	282 (10.3%)
Moderate	1306 (52.8%)	3540 (57.6%)	1389 (50.6%)
Severe	236 (9.5%)	1396 (22.7%)	1075 (39.1%)

### Correlations of pain indicators with the total frailty score

The “pain medication variable” presented a statistically significant moderate positive correlation with frailty (*P* < 0.001; rho = 0.358). Higher levels of frailty were associated with the intake of more pain medication. Similarly, other variables, such as pain level (*P* < 0.001; rho = 0.316) or polypharmacy (*P* < 0.001; rho = 0.327), were statistically significant with frailty with moderate positive correlations.

All the pain locations had a weak and positive correlation with frailty. These associations were all statistically significant (*P* < 0.05 in the mouth/teeth; *P* < 0.001 in the rest), except for the variable, “localized pain in the back,” which did not have a significant association with frailty (rho = −0.004, *P* = 0.666).

### Multivariate associations among frailty classification with pain indicators

A multinomial logistic regression has been estimated to associate, in a multivariate model, frailty with the pain indicators while controlling for age, sex and country of residence. This multinomial logistic regression associates being prefrail versus robust and being frail versus robust using these indicators: taking drugs for pain in the joint; taking drugs for other pain; location of pain (back, hips, knees, other joints, mouth/teeth, others parts that are not joints or all over the body); taking five drugs or more per day; level of pain (dummy coded as mild vs severe, and moderate vs severe); age; sex; and country of residence (dummy coded with Spain as the reference country).

The fit of the overall model was assessed first with a likelihood ratio test (χ^2^) that compares the fit of our model with a null model (no indicator), with a significant test indicating that indicators do a relevant job of explaining variance in frailty (χ^2^ (38) = 2828.22, *P* < 0.001). Second, Pearson and deviance tests were also carried out, and a non‐significant result points out a good model fit. Indeed, both Pearson and deviance tests were non‐significant: *P* = 0.065 and *P* = 1, respectively. Finally, a pseudo‐R‐squared was calculated to estimate the percentage of variance explained by the model. Nagelkerke's pseudo‐R squared was 0.275, showing that all the indicators in the multinomial logistic regression jointly explain 27.5% of the variance.

Once the overall fit had been established, the impact of the indicators on the probability of being prefrail versus being robust was calculated. These estimates are presented in Table 3, and can be consulted in depth there. Nevertheless, some results are worth noting. Among the pain indicators, only pain in other joints and in the mouth/teeth were not statistically significant. Among those statistically significant, the main associated indicators were pain all over the body and level of pain. Those participants with pain all over the body were 3.30‐fold (see the odds ratio in Table 3) more likely to be prefrail than robust, whereas those with severe versus mild pain were 2.38‐fold more likely to be prefrail than robust, and those with severe versus moderate pain were 1.63‐fold more likely to be prefrail rather than robust. Regarding the control variables, all were significantly related to prefrailty status. An increase of 1 year in age made 6% more likely to be prefrail versus being robust. Female participants were 36% more likely to be classified as prefrail rather than robust. Finally, and with respect to country differences, the series of dummy indicators, with Spain as the reference country, showed that Spaniards were more likely to be prefrail when compared with Swedish, French and Czechs, whereas Greeks were more likely to be prefrail than Spaniards. Also, Estonians and Spaniards had no statistically significant differences.

**Table 3:** Multinomial logistic regression results to predict frailty classification

Variable	Prefrail vs robust			Frail vs robust		
	B	Odds and 95% CI	*P*	B	Odds and 95% CI	*P*
Intercept	−3.184	—	<0.001	−7.352	—	<0.001
Drugs for						
Joint pain	0.361	1.43 (1.26, 1.63)	<0.001	0.487	1.63 (1.39, 1.89)	<0.001
Other pain	0.380	1.46 (1.27, 1.67)	<0.001	0.516	1.67 (1.43, 1.95)	<0.001
Location of pain						
Back	0.143	1.15 (1.02, 1.30)	0.020	0.127	1.13 (0.98, 1.31)	0.088
Hips	0.389	1.47 (1.27, 1.70)	<0.001	0.629	1.87 (1.58, 2.21)	<0.001
Knees	0.410	1.51 (1.33, 1.70)	<0.001	0.517	1.68 (1.55, 1.94)	<0.001
Other joints	0.098	1.10 (0.96, 1.25)	0.136	0.231	1.26 (1.08, 1.47)	0.003
Mouth/teeth	0.242	1.27 (0.86, 1.98)	0.227	0.338	1.40 (0.88, 2.21)	0.148
Other parts not joint	0.345	1.41 (1.20, 1.65)	<0.001	0.604	1.83 (1.52, 2.19)	<0.001
All over	1.196	3.30 (2.40, 4.55)	<0.001	1.547	4.69 (3.31, 6.67)	<0.001
Taking 5 drugs a day	−0.199	0.82 (0.79, 0.84)	<0.001	−0.357	0.70 (6.67, 0.72)	<0.001
Level of pain						
Mild vs severe	−0.858	0.42 (0.35, 0.51)	<0.001	−1.61	0.19 (0.16, 0.25)	<0.001
Moderate vs severe	−0.497	0.61 (0.51, 0.72)	<0.001	−1.00	0.36 (0.30, 0.44)	<0.001
Age	0.063	1.06 (1.05, 1.07)	<0.001	0.110	1.11 (1.10, 1.12)	<0.001
Sex (female vs male)	0.314	1.36 (1.32, 1.53)	<0.001	0.535	1.71 (1.48, 1.96)	<0.001
Country						
Sweden vs Spain	−0.555	0.57 (0.47, 0.69)	<0.001	−0.968	0.38 (0.29, 0.48)	<0.001
France vs Spain	−0.223	0.80 (0.66, 0.96)	0.019	−0.182	0.83 (0.66, 1.04)	0.113
Greece vs Spain	0.418	1.51 (1.21, 1.89)	<0.001	0.680	1.97 (1.53, 2.53)	<0.001
Czech Republic vs Spain	−0.395	0.67 (0.56, 0.79)	<0.001	−0.421	0.66 (0.53, 0.80)	<0.001
Estonia vs Spain	0.115	1.12 (0.94, 1.33)	0.198	0.182	1.20 (0.97, 1.47)	0.087

B, regression slope; CI, Confidence Interval; Odds, odds ratio.

The estimated impacts of the indicators on the probability of being frail versus robust are presented in Table 3. Again, some results are worth noting. Among the pain indicators, only pain in the back and in the mouth/teeth were not statistically significant. Among the statistically significant pain indicators, the main predictors were again pain all over the body and level of pain. Participants with pain all over the body were 4.69‐fold (see the odds ratio in Table 3) more likely to be frail than robust, whereas those with severe versus mild pain were 5.02‐fold more likely to be frail than robust, and those with severe versus moderate pain were 2.71‐fold more likely to be frail rather than robust. Regarding the control variables, all were significantly related to frailty status. Every increase of 1 year in age made 12% more likely to be frail versus being robust. Female participants were 71% more likely to be classified as frail rather than robust. Regarding country differences, the series of dummy indicators, with Spain as the reference country, showed that Spaniards were more likely to be frail when compared with Swedish and Czechs, whereas Greeks were more likely to be frail than Spaniards. Additionally, Estonians, French and Spaniards presented no statistically significant differences.

## Discussion

Pain and frailty are complex and interlinked challenges faced by older adults.^32^ The pathophysiological changes and pain‐induced reduction in physical activity could be important precipitants of frailty and consequent adverse outcomes. Additionally, addressing pain concerns is crucial, as they could affect the person's state of mind, inducing hypervigilance of pain, catastrophism and kinesiophobia, increasing functional limitations of prefrail and frail individuals.^33^ Therefore, understanding these links is critically important for the optimization of medical care for older adults.

To our knowledge, this is the first study with a large sample collected from different European countries that analyzes different indicators of pain and its association with physical frailty in a multivariate context. This study showed that certain characteristics of pain, particularly widespread pain, and its severity were strongly associated with prefrailty and frailty levels. Across all the countries studied, older age and being female consistently correlated with a higher risk of prefrailty and frailty. However, there were variations between countries, with Spaniards having a higher likelihood of prefrailty and frailty compared with individuals from Sweden, France and the Czech Republic. Greeks, in contrast, showed increased odds ratios compared with Spaniards. Together, these indicators accounted for 27.5% of the differences observed in frailty levels across the study population.

Regarding the location of pain, the present study identified a significant association between widespread pain and frailty, consistent with previous research. Pain in multiple body locations is more common among frail older adults,^34^ and generalized chronic musculoskeletal pain has been linked to frailty.^9^ Our findings also showed that joint pain in the lower limbs, especially in the knees, is associated with frailty, supporting earlier studies.^35^, ^36^ Longitudinal studies further show that pain related to osteoarthritis in the lower limbs increases the risk of developing frailty, with knee pain, particularly bilateral pain, being strongly associated with a higher risk of prefrailty and frailty.^37^, ^38^ It can be extrapolated that frail older adults will be at higher risk and more susceptible to pain in these locations. Irrespective of frailty status, other studies examining pain have also reported that multisite pain is common among older adults and women.^10^, ^39^ Understanding these commonly reported areas of pain and sex differences can help healthcare professionals to carry out focused assessments, and to prescribe targeted exercises and therapies.

Regarding pain medication, the study by Blyth *et al*. showed that opioid use varied with participants' frailty status, with 6.5% of frail individuals using opioids compared with 1.2% of robust individuals.^40^ This finding is consistent with the present study, where the use of pain medication was positively associated with the level of frailty. Similarly, other studies have also found a correlation between frailty and the use of analgesics.^41^

Unexpectedly, in this multivariate context, polypharmacy was negatively associated with prefrailty and frailty. According to the present findings, this means that, within the context of pain and frailty, individuals with polypharmacy were less likely to be frail. In contrast, different authors suggest a positive association between polypharmacy and frailty in older adults.^42^ A possible reason for our differing results could be the concept of polypharmacy used. In the present study, polypharmacy was assessed by asking if participants took at least five medications, including dietary supplements. This approach, which includes supplements, such as vitamins and minerals with protective effects or a low threshold of medications, might have influenced our findings. In this line, Moulis *et al*. found that a cut‐off of six drugs distinguished frailty status in French participants aged ≥65 years.^43^ Likewise, Gnjidic *et al*. identified 6.5 drugs as the optimal threshold in Australian men aged ≥70 years.^44^ A recent review suggests using this threshold or the mean number of drugs to standardize the definition of polypharmacy in future studies.^42^ Additionally, it is also worth considering that the relationship between frailty and the use of a larger number of medications was not significant in some complex multivariate regression models, where other variables in the model become more relevant to the development of frailty than medication exposure itself.^45^, ^46^

Furthermore, the present findings showed a strong correlation between pain levels and increased frailty, aligning with previous research. Chiou *et al*. reported that individuals with moderate to severe pain were threefold more likely to be frail, highlighting a global phenomenon that transcends races, ethnicities and cultures.^32^ Similarly, a longitudinal study by Rodríguez‐Sánchez *et al*. found that pain intensity was linked to a higher risk of frailty, even after accounting for various conditions.^47^ This information could significantly influence the development of prevention strategies aimed at mitigating frailty.

In line with the literature, the present findings showed that in the model analyzed, older age and female sex were consistently associated with higher odds ratios for prefrailty and frailty status.^48^, ^49^ Evidence also shows differences in pain between men and women, with women showing greater vulnerability to pain. Epidemiological studies confirmed varying pain prevalence across countries and settings, with pain being more prevalent in women.^50^, ^51^ These differences might be linked to biological factors, such as genetics, sex hormones and physiology, as well as psychological and sociocultural factors, including beliefs and expectations. An approach on how sex/gender differences influence pain‐related cognitive–emotional processing could also contribute to these differences.^52^

Our data showed that individuals from Spain are significantly more prone to prefrailty and frailty compared with other studied populations. This aligns with the findings of Theou *et al*. that higher‐income countries, such as Sweden, show lower rates of frailty and mortality risk.^53^ This observation clarifies why Spain, despite being a higher‐income country, still experiences higher incidence rates compared with other countries, such as Sweden. In addition to considering economic characteristics, healthcare systems and genetic factors, other determinants could also be taken into account. Existing literature suggests that joint pain could be exacerbated by higher levels of temperature and humidity, particularly in older adults.^54^ Given that southern countries, such as Spain, have warmer climates compared with northern countries, it is plausible that these environmental factors could contribute to the heightened perception and spread of pain among Spaniards. Castell *et al*. carried out a study involving 2455 individuals aged 65–85 years from six European countries.^55^ Their findings showed a clear contrast in the prevalence of joint pain between Spaniards and Greeks. This discrepancy highlights the need to further explore how climatic and geographic factors might affect pain and frailty differently across countries. Additionally, neuropsychological factors might mediate the relationship between pain and frailty.^32^, ^56^ It would be valuable to consider these factors when examining the differences between countries. The literature emphasized that the perception of pain severity is subjective and influenced by various factors, including psychological factors, such as anxiety or depression.^57^ This observation is supported by recent evidence highlighting the prevalence of depression across different European countries, where neither Sweden, France nor the Czech Republic ranked among the top countries with the highest prevalence of depression.^58^ In addition, among the countries analyzed, Spain and Greece show the highest prevalence of late‐life depression in the European region. The factors most strongly associated with depression include being troubled by pain, the number of chronic diseases, limitations in activities of daily living, reduced grip strength and cognitive impairment.^59^ It would be valuable for future studies to further investigate the influence of neuropsychological factors in this context of pain and frailty to better understand the differences observed between countries.

The main limitation of the present study was that most of the variables were self‐reported by the participants, except for grip strength, which was measured with a dynamometer, so the data could be subjected to bias. However, the sample size was robust and significantly larger than other studies on the topic. Additionally, the sample also covered people from different countries in Europe. A more detailed analysis of the sample from each country and proper evaluation of potential confounders would be needed.

There are significant associations between frailty and pain indicators in a sample of community‐dwelling older people from various European countries. Pain indicators, particularly pain all over the body and the severity of pain, emerged as significant indicators for both prefrailty and frailty status, highlighting the impact of pain on frailty development. Additionally, country‐specific differences were observed, with variations in frailty prevalence across different populations. Advancing age and female sex were consistently linked to increased odds ratio of prefrailty and frailty, emphasizing their role as key determinants of frailty in the analyzed model. These findings provide valuable insights into the complex interplay between pain indicators and frailty. Understanding these links could be useful when developing strategies to prevent or treat frailty in older adults through the use of targeted pain management techniques.

## Disclosure statement

The authors declare no conflict of interest.

## Data Availability

The data that support the findings of this study are available on request from the corresponding author. The data are not publicly available due to privacy or ethical restrictions.
